# Effects of Percutaneous Electrical Nerve Stimulation on Countermovement Jump and Squat Performance Speed in Male Soccer Players: A Pilot Randomized Clinical Trial

**DOI:** 10.3390/jcm10040690

**Published:** 2021-02-10

**Authors:** Gracia María Gallego-Sendarrubias, José Luis Arias-Buría, Edurne Úbeda-D’Ocasar, Juan Pablo Hervás-Pérez, Manuel Antonio Rubio-Palomino, César Fernández-de-las-Peñas, Juan Antonio Valera-Calero

**Affiliations:** 1 Department of Physical Therapy, Universidad Camilo José Cela, Villanueva de la Cañada, 28692 Madrid, Spain; gmgallego@ucjc.edu (G.M.G.-S.); eubeda@ucjc.edu (E.Ú.-D.); jphervas@ucjc.edu (J.P.H.-P.); 2 Department of Physical Therapy, Occupational Therapy, Rehabilitation and Physical Medicine, Universidad Rey Juan Carlos, 28922 Alcorcón, Spain; joseluis.arias@urjc.es (J.L.A.-B.); cesar.fernandez@urjc.es (C.F.-d.-l.-P.); 3 Cátedra Institucional en Docencia, Clínica e Investigación en Fisioterapia, Terapia Manual, Punción Seca y Ejercicio Terapéutico, Universidad Rey Juan Carlos, 28922 Madrid, Spain; 4 Private Professional Practice, 28008 Madrid, Spain; 4manuel92@gmail.com

**Keywords:** Percutaneous Electrical Nerve Stimulation, soccer, performance

## Abstract

It has been suggested that Percutaneous Electrical Nerve Stimulation (PENS) can increase muscle strength. No previous study has investigated changes in performance in semiprofessional soccer players. This study compares the effects of adding two sessions of PENS to a training program versus the single training program over sport performance attributes (e.g., jump height and squat speed) in healthy soccer players. A cluster-randomized controlled trial was conducted on twenty-three semiprofessional soccer players who were randomized into an experimental (PENS + training program) or control (single training program) group. The training program consisted of endurance and strength exercises separated by 15-min recovery period, three times/week. The experimental group received two single sessions of PENS one-week apart. Flight time and vertical jump height during the countermovement jump and squat performance speed were assessed before and after each session, and 30 days after the last session. Male soccer players receiving the PENS intervention before the training session experienced greater increases in the flight time, and therefore, in vertical jump height, after both sessions, but not one month after than those who did not receive the PENS intervention (F = 4.289, *p* = 0.003, η 2 p: 0.170). Similarly, soccer players receiving the PENS intervention experienced a greater increase in the squat performance speed after the second session, but not after the first session or one month after (F = 7.947, *p* < 0.001, η 2 p: 0.275). Adding two sessions of ultrasound-guided PENS before a training strength program improves countermovement jump and squat performance speed in soccer players.

## 1. Introduction

Soccer is a popular, high-demanding, and complex team sport where success depends on the performance of ten players and a goalkeeper [1,2]. The physiological demands of this sport are complex since the nature of the exercise pattern is intermittent. For instance, during a soccer match, changes in both the speed of movement (there is a wide scale of speed intensity from walking to sprinting) and direction (e.g., attacking, defending, or position game demands non-linear movement directions) are needed; therefore, players need strong and flexible muscles to success their technical actions (e.g., passing, shooting) [3]. Despite tactical and psychological features are highly important to determine soccer performance [4], some studies had evaluated different physical performance characteristics to predict the player’s success in a match situation.

Although several technical tests have been used to discriminate the performance of soccer players [5,6], Ryman-Augustsson et al. [7] recently reported that some physical performances (e.g., sprint velocity, and jump height) are associated with greater soccer professional success. Since these physical features could increase the chances of professional soccer success, several studies recommend maximal strength training to obtain favorable effects on 1RM, sprint, and jumping performance in male football players [8,9]. Thus, the capacity to increase the maximal strength in half squats determines sprint performance and jumping height in high-level football players [10].

Percutaneous Electrical Nerve Stimulation (PENS) is a therapeutic approach that has been used for pain management of migraine [11], nerve analgesia in upper [12,13], and lower [14] extremities, and back pain [15]. Although most previous studies have focused on pain management, others have investigated the effects of PENS in other outcomes. For instance, Álvarez-Prats et al. have recently reported an increase in maximal quadriceps muscle strength after applying low-frequency PENS in the femoral nerve [16]. Similarly, de-la-Cruz-Torres et al. have also shown that applying PENS is able to increase hamstring flexibility in healthy people [17]. To the best of the authors’ knowledge, no study has investigated the effects of PENS targeting the femoral nerve to improve jump height or squat speed in soccer players. Therefore, the current randomized clinical trial aims to compare the effects of adding two sessions of PENS to a specific training program versus the single training program over sport performance attributes (e.g., jump height and squat speed) in healthy soccer players. We hypothesized that soccer players receiving PENS in addition to a specific training program would experience better outcomes than those receiving just the specific training program.

## 2. Material and Methods

### 2.1. Study Design

A randomized, parallel-group, controlled, single-blind, cluster-randomized clinical trial comparing the inclusion of two sessions of low-frequency PENS over the femoral nerve to a specific training program versus just a specific training program in soccer players was conducted. The primary outcome of the study was one-month changes in jump height as assessed by a countermovement jump (CMJ). The secondary outcome was the squat performance speed. This clinical trial followed the Consolidated Standards of Reporting Trials (CONSORT) for pragmatic clinical trials [18]. This study was conducted according to the Declaration of Helsinki, approved by the Institutional Ethics Committee of Clinical Research of Alfonso X el Sabio University (UAX 20-02-2020), and prospectively registered at ClinicalTrials.gov with the registration number NCT04427553.

### 2.2. Participants

A consecutive sample of semiprofessional soccer players was screened for eligibility criteria from June 2020 to October 2020 from two semiprofessional clubs located in Spain Participants had to be male semiprofessional soccer players, aged from 18 to 40 years old, with a training frequency of at least three days per week. Exclusion criteria included needle fear, prior lower extremity or spine surgery, presence of pain the previous month, any musculoskeletal or neuropathic condition, or any contraindication for needling treatment (e.g., anticoagulant).

### 2.3. Randomization and Masking

Participants were randomly assigned to the experimental (PENS + training program) or control (single training program) group. Concealed allocation was conducted by using a random-number generator (Research Randomizer Vr.4.0). Individual and sequentially numbered cards with the random assignment were folded in sealed opaque envelopes. One external researcher selected the envelope and proceeded with appropriate allocation. Then, the participants’ allocation was revealed after baseline data collection. The rater was blinded to the allocation group.

### 2.4. Percutaneous Electrical Nerve Stimulation

Participants within the experimental group received two sessions of low-frequency PENS, once per week, performed by an experienced clinician with 10 years of experience in this procedure. The ultrasound-guided PENS intervention consisted of the bilateral application of a biphasic asymmetric compensated electrical current with a rectangular positive phase and a negative triangular phase, at a frequency of 10 Hz, a pulse width of 240 µs and maximal intensity allowed over the motor threshold and bellowed the pain threshold of the subject. The electrical current was applied throughout a Physio Invasiva^®^ CE0120 (Prim Fisioterapia y Rehabilitación, Madrid, Spain) device, with 0.30 × 10 mm Agupunt APS^®^ needle.

**Figure 1:**
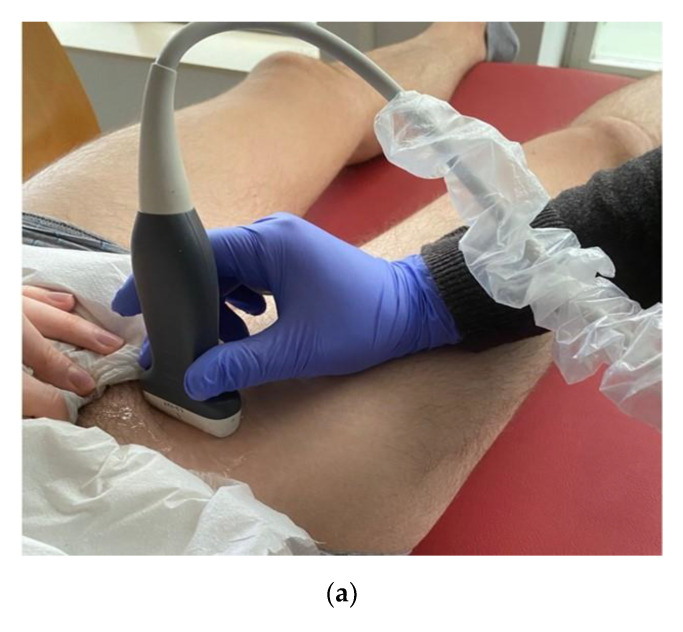

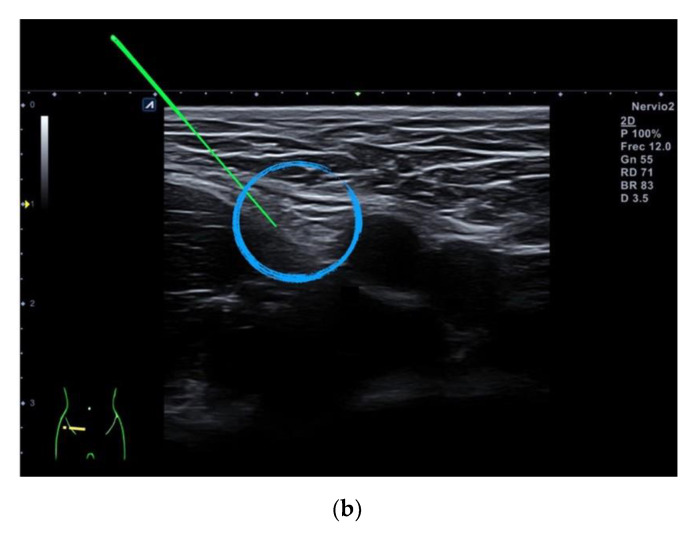
Femoral nerve ultrasound location for applying PENS over the left lower extremity: (**a**) Transducer placement; (**b**) ultrasound imaging of the femoral nerve.

The peripheral nerve selected was the femoral, located over the femoral triangle with an Alpinion Ecube i8 ultrasound equipment and an E8-PB-L3-12T 3-12MHz linear probe in a transverse section (Figure 1a). After cleaning the skin with chlorhexidine (Lainco^®^ 2%), the needle was emplaced using an in-plane approach, with a 45° angle to the skin surface, until reaching the epineurium of the femoral nerve at its lower and lateral aspect (Figure 1b). The axonal topography of the femoral nerve refers to this site as the best part of the nerve to locate the motor axons of the quadriceps muscle [19]. The intervention was applied bilaterally to both lower extremities.

The procedure evoked pain-free maximal muscle contractions in 10 sequences of 10 s, each one with 10 s rest period between series according to Álvarez-Prats et al. [16] protocol.

### 2.5. Training Program

The training program for both groups consisted of endurance and strength training separated by 15-min recovery period, three times a week, considering the specific routine training recommendations reported by Makhlouf et al. for soccer players [20]. Participants performed 4 × 4 min bouts at 90–95% of maximal heart rate interspersed with 3 min jogging at 70% of maximal heart rate and four sets of four repetitions of half-squats, sprint performance, and jumping height [21].

### 2.6. Outcomes

Outcomes were evaluated before and immediately after each PENS session (5 min after applying PENS), and 30 days after the second session by an assessor blinded to the subject allocation group.

**Figure 2:**
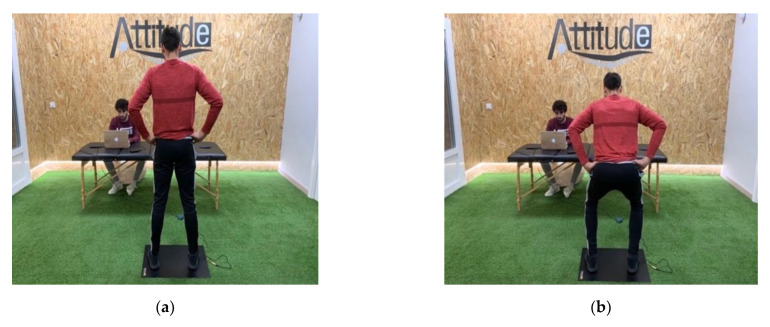
Countermovement jump measurement: (**a**) Starting position; (**b**) vertical jump after a fast-down countermovement.

The primary outcome measure was countermovement jump. The jump started in an upright position with participants hands in their waist (Figure 2a). Athletes performed a vertical jump after a fast-down countermovement (Figure 2b). During the knees and hips flexion, the trunk remained the most upright as possible. All participants performed three jumps without feedback, and the mean value of the three attempts was calculated for the analysis. The countermovement jump was measured using a Chronopump-Boscosystem DIN-A2 contact platform, obtaining a score of flight time (milliseconds) [22]. This platform exhibited a trivial standardized typical error of estimate (0.001 ms), as well as minimal variability (coefficient of variation 0.01%) [22]. Rago et al. had reported that the standard error of measurement (SEM) of the force platform was 0.004 ms and that the smallest worthwhile change (SWC) can be considered with 0.006 ms [23]. In addition, jump height was also calculated as the height of the center of mass displacement calculated from integration (0.001 s time constant) of the vertical ground reaction force and the measured body mass [24]. Based on data from Pueo et al., changes of 0.09 cm in jumping height could be considered clinically relevant with the Chronopump-Boscosystem DIN-A2 contact platform [22].

**Figure 3:**
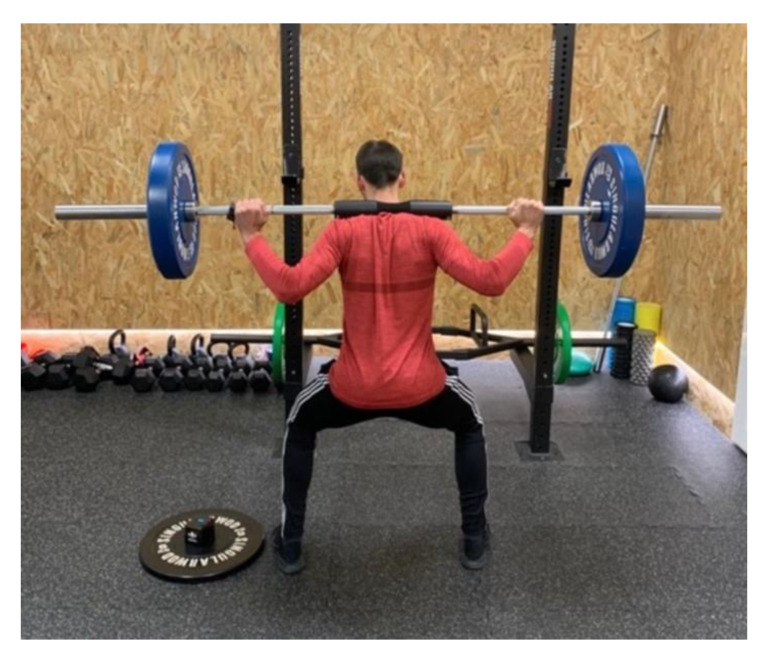
Squat performance speed assessment.

The secondary outcome was the squat performance speed as assessed by using the Speed4Lift device. The reliability estimates for the Speed4Lift device had been calculated with different loads ranging from 45% to 85% 1RM and have shown good-to-excellent reliability (ICC 0.81–0.94; coefficient of variation: 2.42–3.92%), Pérez-Castilla et al., 2019 [25]. All squats were performed using a 20 kg Olympic bar with two 20 kg discs on each side. The soccer player placed the bar over their shoulders in a 90° triple-flexion position (Figure 3). The measurements were performed during the concentric contraction phase.

### 2.7. Treatment Side Effects

Participants were asked to report any adverse event experienced during or after the interventions (up to the 1-month duration of this study). Adverse events were defined as sequelae of short-medium term symptoms perceived as unacceptable to the patient and required further treatment by using a self-reported document provided to the participants and informed to an external clinician during the study [26].

### 2.8. Sample Size Determination

The sample size was calculated using Ene 3.0 software (Autonomic University of Barcelona, Barcelona, Spain). The calculation was based on detecting a between-groups difference of 0.01 ms on flight time on the countermovement jump, assuming a standard deviation of 0.006 ms [23], a 2-tailed test, an alpha level (α) of 0.05, and the desired power (β) of 90%. The estimated desired sample size was calculated to be 10 individuals per group.

### 2.9. Statistical Analysis

All statistical analyses were performed in IBM SPSS Statistics Version 22 (IBM Corporation, Armonk, NY, USA), setting at a significance level *p* < 0.05. Descriptive statistics were used to characterize the sample, assess the distribution, and summarize the variables. Normal-distributed data were described by means, standard deviation (SD), and 95% Confidence Intervals (95% CI). Non-normal distribution data were descriptively presented as median and interquartile range. The distribution was verified by histograms, a Shapiro–Wilk test for normality distribution, and a Levene test for variance homogeneity. Between-groups comparability at baseline was assessed with independent t-test or Mann-Whitney U-test for continuous data and chi-square tests of independence for categorical data. Analysis of Covariance (ANCOVA) using baseline values as covariates has been shown to be more powerful than repeated measures analysis of variance (RM ANOVA) when random group assignment is used [27]. Therefore, a 2 × 2 ANCOVA with time (before, after treatment) as the within-subjects factor, group (PENS + training or training alone) as the between-subjects factor, and baseline values as covariates was used to examine the effects of the interventions. Post hoc analyses were conducted with t-Student tests for independent samples. The effect size was calculated when the Partial Eta Squared (η 2 p) was significant. A Partial Eta Squared of 0.01 was considered small, 0.06 medium, and 0.14 large [28].

## 3. Results

**Figure 4:**
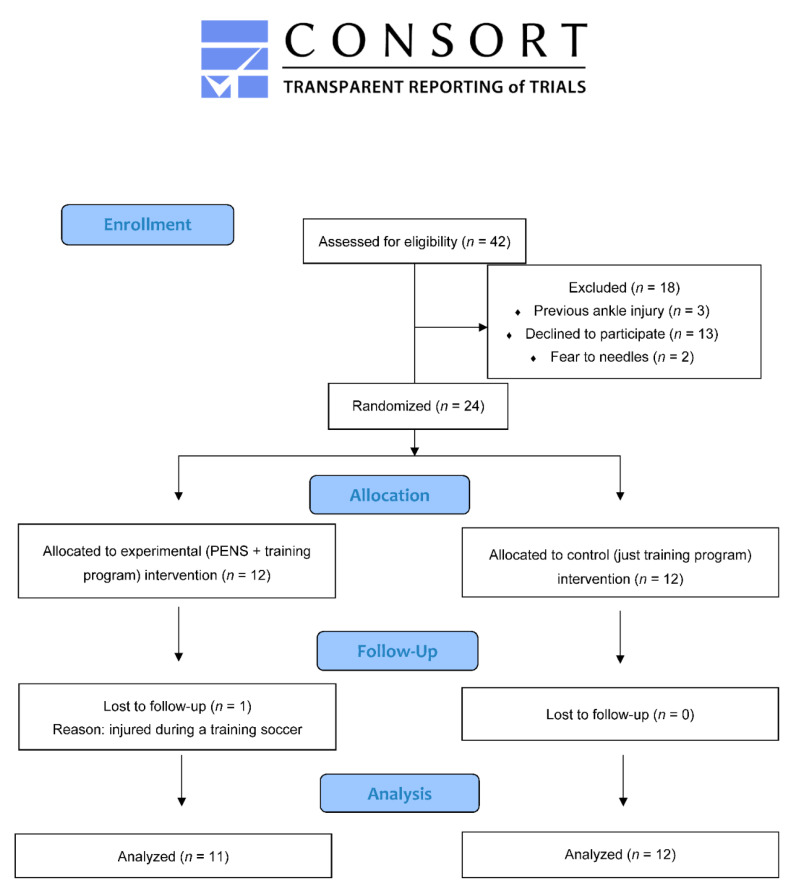
CONSORT 2010 Flow Diagram.

Forty-two (*n* = 42) soccer players were initially recruited in June 2020. Eighteen participants were excluded as follows: Fear of needles (*n* = 2), previous ankle injury (*n* = 3), refuse to participate for personal reasons (*n* = 13). Twenty-four soccer players were finally included and randomized into one of two groups: PENS + training (*n* = 12) or just training (*n* = 12). One participant from the PENS group was lost at follow-up, due to an injury during one training session (Figure 4). Any participant reported adverse effects during the study. Both groups were comparable at baseline (Table 1).

The mixed-model ANCOVA revealed a significant group * time interaction for the countermovement jump (F = 4.289, *p* = 0.003, η 2 p: 0.170): Male soccer players receiving the PENS interventions before the training session experienced greater increases in the flight time, and therefore, in vertical jump height, after both sessions, but not one month after than those who did not receive the PENS intervention (Table 2 and Table 3). Post hoc analyses revealed significant within-session increases in the PENS group and at the one-month follow-up period (all, *p* < 0.01) with no significant within-group changes in the training group.

The mixed-model ANCOVA also revealed a significant group * time interaction for squat performance speed (F = 7.947, *p* < 0.001, η 2 p: 0.275): Male soccer players receiving the PENS interventions before the training session experienced a greater increase in the squat performance speed after the second session, but not after the first session or one month after (Table 4). Post hoc analyses showed significant within-session increases in the PENS group and at the one month follow-up period (all, *p* < 0.01). In addition, significant within-group changes in the training control group were found, but just one month after.

## 4. Discussion

### 4.1. Findings

The current trial assessing the effects of PENS in physical performance in semiprofessional soccer players found that combining just two sessions of ultrasound-guided PENS with a training program improved flight time on a countermovement jump and squat performance speed in semiprofessional soccer players.

The positive effects of PENS for the management of chronic pain are documented in the current literature [29]. Although PENS is a promising intervention in this field, most of the studies are case reports. One high-quality clinical trial found that PENS is more effective than sham PENS for pain management after 12-week treatment in cervical spondylosis [30]. Studies investigating the effects of PENS on motor output are scarce.

According to a previous review [31], the use of PENS could induce significant increases in muscle fiber cross-sectional area, fiber type, isokinetic peak torque, maximal isometric and dynamic strength, and motor performance skills. However, most of the studies assessing the effects of PENS have been conducted in chronic pain populations. A recent study found that PENS could be a potentially effective technique for increasing isometric strength of the quadriceps in inhibited musculature in subjects with unilateral knee pathology [16]. Since therapeutic interventions focusing on the voluntary quadriceps muscle activation could improve the efficacy of exercise for strength improvement [32], PENS could be a useful procedure to improve physical performance to be used combined with exercise. The hypothesis would be that fast-twitch fibers are preferentially stimulated by PENS, and inhibitory influences that are physiologically present during maximal voluntary efforts would be delayed during the application of PENS, providing a more intense contraction of the musculature that is being stimulated during exercise programs. In agreement with this hypothesis, the results of our study suggest that the inclusion of two sessions of PENS before the application of a training program reported short-term improvements in physical performance in semiprofessional soccer players. Similar results were reported by Herrero et al. [33] in physical education students, where a greater strength increase was found when voluntary contractions were combined with the application PENS.

We observed between-groups changes ranging from 0.01 ms to 0.02 ms in flight time, equivalent to changes from 1 cm to 3 cm in vertical jump height. These changes were bigger than the SWC for flight time calculated by Rago et al. (0.006 ms) [23], suggesting that the observed changes could be considered clinically relevant. In fact, Krommes et al. [34] found that applying the 10-weeks Nordic hamstring protocol during the preseason in elite soccer players also improves 2 cm the vertical jump height during the countermovement jump. It seems that short-term changes found after applying just two sessions of PENS could improve physical performance in soccer players. Future studies implementing this intervention during regular seasons are required.

Current evidence shows a strong correlation between jumping performance in elite soccer players with maximal strength in half squats and sprinting (0–30 m sprints, as well as the 10 m shuttle run test), and a maximal strength improvement based on a training regimen with few repetitions, high loads, highlighting the maximal mobilization of force during the concentric phase of the half squat to improve sprint and jumping performance [10]. PENS could be an interesting therapeutic strategy to improve physical performance features associated with jumping performance (e.g., sprinting, and maximal strength) in the short term in preparation to exercise programs. In addition, these improvements could be particularly beneficial for soccer players, since anterior cruciate ligament (ACL) injures are commonly found in this cohort [35], and strengthening programs with plyometric training are recommended to reduce their risk [36].

### 4.2. Limitations

Although the results of this study are promising, some potential limitations should be recognized. First, muscular electromyographic contraction activity was not assessed. The physical performance improvement observed after treatment could be correlated with fiber recruitment capacity changes. Second, the generalizability of the results should be applied just to male semiprofessional soccer players. Finally, the sample was relatively small, and further research with a greater sample is needed. Future studies should explain the mechanisms underlying the observed improvement and determine the effects of PENS in other sports players, including females.

## 5. Conclusions

This study showed that combining two sessions of low-frequency PENS to the femoral nerve with a training program produced greater improvements in flight time (vertical jump height) on the countermovement jump than the strength training program alone at short-term (immediately after interventions and up to seven days), but not one month after. Changes in squat performance speed were only observed after the second session.

Our results suggest that PENS could be a complementary intervention to exercise for improving physical performance in sports players.

## Figures and Tables

**Table 1:** Participants’ characteristics at baseline.

Outcomes	PENS + Training Group	Training Group
Age (years)	26 ± 4.5	25 ± 4.0
Weight (kg)	73.5 ± 5.5	74.5 ± 9.5
Height (m)	1.8 ± 0.05	1.8 ± 0.1
BMI (kg/cm^2^)	23.5 ± 1.95	23.5 ± 2.9
Countermovement Jump Height (cm)	31.5 ± 3.5	32.0 ± 5.0
Countermovement Jump Flight Time (s)	0.507 ± 0.029	0.509 ± 5.0
Squat Performance Speed (m/s)	0.55 ± 0.3	0.6 ± 0.3

Values are Mean ± Standard Deviation (SD).

**Table 2:** Timeline scores, within-group, and between-group changes of flight time (s) during the Countermovement Jump (CMJ).

	PENS + Training Group (*n* = 11)	Training Group (*n* = 12)	Within-Group Differences						Between-Groups Differences		
			PENS + Training			Training Group					
CMJ Flight Time (s)			Immediate Differences	Baseline/Session 2	Baseline/Follow-Up	Immediate Differences	Baseline/Session 2	Baseline/Follow-Up	Immediate Differences	Baseline/Session 2	Baseline/Follow-Up
Before Session 1 (Day 1)	0.507 ± 0.029 (0.487, 0.526)	0.509 ± 0.041 (0.482, 0.534)	0.016 ± 0.012 (0.008, 0.024) 0.001	0.017 ± 0.006 (0.012, 0.021) <0.001	0.010 ± 0.008 (0.004, 0.015) 0.003	−0.001 ± 0.008 (−0.006, 0.004) 0.620	0.005 ± 0.016 (−0.005, 0.015) 0.299	0.008 ± 0.020 (−0.004, 0.020) 0.184	0.017 ± 0.004 (0.008, 0.026) <0.001	0.011 ± 0.005 (0.001, 0.022) 0.032	0.001 ± 0.006 (−0.011, 0.015) 0.792
After Session 1 (Day 1)	0.523 ± 0.031 (0.502, 0.544)	0.507 ± 0.041 (0.480, 0.533)									
Before Session 2 (Day 7)	0.510 ± 0.029 (0.490, 0.528)	0.522 ± 0.046 (0.492, 0.551)	0.014 ± 0.009 (0.008, 0.020) <0.001			−0.008 ± 0.010 (−0.014, −0.001) 0.017			0.022 ± 0.003 (0.014, 0.030) <0.001		
After Session 2 (Day 7)	0.524 ± 0.030 (0.503, 0.544)	0.514 ± 0.048 (0.482; 0.544)									
Follow-up (after 30 days)	0.517 ± 0.030 (0.496, 0.545)	0.517 ± 0.045 (0.487, 0.545)									

Data are expressed as mean ± SD (95% CI), with their *p* value.

**Table 3:** Timeline scores, within-group, and between-group changes of the vertical jump height during Countermovement jump (CMJ).

	PENS + Training Group (*n* = 11)	Training Group (*n* = 12)	Within-Group Differences						Between-Groups Differences		
			PENS + Training			Training Group					
CMJ (cm)			Immediate Differences	Baseline/Session 2	Baseline/Follow-Up	Immediate Differences	Baseline/Session 2	Baseline/Follow-Up	Immediate Differences	Baseline/Session 2	Baseline/Follow-Up
Before Session 1 (Day 1)	31.5 ± 3.5 (29.0, 34.0)	32.0 ± 5.0 (29.0, 35.0)	2.0 ± 1.5 (1.0, 3.0) <0.001	2.5 ± 1.04 (1.5, 3.5) <0.001	1.5 ± 1.0 (0.5, 2.5) 0.002	−0.5 ± 1.0 (−1.6, 0.6) 0.645	0.5 ± 2.5 (−0.5, 1.5) 0.257	0.7 ± 2.0 (−0.6, 2.0) 0.174	2.5 ± 0.5 (1.0, 4.0) 0.001	2.0 ± 1.0 (0.5, 3.5) 0.043	0.8 ± 0.8 (−1.0, 2.6) 0.828
After Session 1 (Day 1)	33.5 ± 4.0 (31.0, 36.0)	31.5 ± 5.0 (28.5, 34.5)									
Before Session 2 (Day 7)	32.0 ± 3.5 (29.5, 34.50)	33.5 ± 6.0 (30.0, 37.0)	2.0 ± 1.0 (1.0, 3.0) <0.001			−1.00 ± 1.5 (−2.5, 0.5) 0.615			3.0 ± 0.5 (1.8, 4.2) <0.001		
After Session 2 (Day 7)	34.0 ± 4.0 (31.0, 37.0)	32.5 ± 6.0 (28.5, 36.5)									
Follow-up (after 30 days)	33.0 ± 3.5 (30.5, 35.5)	33.0 ± 5.5 (29.5, 36.5)									

Data are expressed as mean ± SD (95% CI), with their *p* value.

**Table 4:** Timeline scores, within-group, and between-groups changes in squat performance speed (SPS).

	PENS + Training Group (*n* = 11)	Training Group (*n* = 12)	Within-Group Differences						Between Groups Differences		
			PENS + Training			Training Group					
SPS (m/s)			Immediate Differences	Baseline/Session 2	Baseline/Follow-Up	Immediate Differences	Baseline/Session 2	Baseline/Follow-Up	Immediate Differences	Baseline/Session 2	Baseline/Follow-Up
Before Session 1 (Day 1)	0.55 ± 0.3 (0.35, 0.75)	0.6 ± 0.3 (0.4, 0.8)	0.35 ± 0.3 (0.15, 0.55) 0.004	0.35 ± 0.3 (0.2, 0.6) 0.001	0.35 ± 0.3 (0.15, 0.55) 0.003	0.1 ± 0.3 (−0.1, 0.3) 0.279	0.15 ± 0.35 (−0.05, 0.35) 0.151	0.25 ± 0.25 (0.1, 0.4) 0.008	0.2 ± 0.15 (−0.05, 0.45) 0.98	0.2 ± 0.15 (0.0, 0.4) 0.07	0.1 ± 0.1 (−0.15, 0.4) 0.483
After Session 1 (Day 1)	0.9 ± 0.2 (0.7, 1.1)	0.7 ± 0.3 (0.5, 0.9)									
Before Session 2 (Day 7)	0.7 ± 0.1 (0.6, 0.8)	0.9 ± 0.25 (0.7, 1.1)	0.25 ± 0.15 (0.15, 0.35) <0.001			−0.15 ± 0.15 (−0.3, 0.0) 0.07			0.4 ± 0.05 (0.2, 0.6) <0.001		
After Session 2 (Day 7)	1.0 ± 0.1 (0.9, 1.1)	0.75 ± 0.25 (0.6, 0.9)									
Follow-up (after 30 days)	0.9 ± 0.1 (0.8, 1.0)	0.85 ± 0.15 (0.75, 0.95)									

Data are expressed as mean ± SD (95% CI), with their *p* value.
